# Boosting CO_2_ and benzene adsorption through π-hole substitution in β-diketonate Cu(ii) complex within non-porous adaptive crystals†

**DOI:** 10.1039/d4ra08463b

**Published:** 2025-02-25

**Authors:** Yoshinori Ikumura, Tadashi Kawasaki, Yuki Ishida, Hirotomo Usui, Sayaka Uchida, Kazuki Kamata, Mikihiro Nomura, Akiko Hori

**Affiliations:** a Graduate School of Engineering and Science, Shibaura Institute of Technology Fukasaku 307, Minuma-ku Saitama 337-8570 Japan ahori@shibaura-it.ac.jp; b Department of Basic Sciences, School of Arts and Sciences, The University of Tokyo 3-8-1 Komaba, Meguro-ku Tokyo 153-8902 Japan; c Graduate School of Engineering and Science, Shibaura Institute of Technology Toyosu 3-7-5, Koto-ku Tokyo 135-8548 Japan

## Abstract

The effect of quadrupole moments in non-porous adaptive crystals of fully, partially, and non-fluorinated β-diketonate Cu(ii) complexes on CO_2_ and hydrocarbon adsorption was systematically investigated using structurally similar models with distinct electronic properties. The fully fluorinated complex significantly enhanced CO_2_ adsorption, particularly at low pressures (<0.1 *P*/*P*_0_), achieving a 1 : 1 stoichiometric ratio through quadrupole interactions, where the positively polarized regions of electrostatic potentials (ESPs) on the Cu(ii) center and pentafluorophenyl rings facilitated CO_2_ binding *via* its quadrupole nature. The perfluorinated complex also exhibited a stepwise vapor adsorption of benzene (C_6_H_6_), exhibiting distinct hysteresis and a 1 : 3 stoichiometric ratio, driven by M⋯π and π-hole⋯π interactions. In contrast, the partially fluorinated complex and non-fluorinated [Cu(dbm)_2_] (dbm = dibenzoylmethanido^−^) showed significantly reduced adsorption capabilities, reflecting the critical role of quadrupole moments and charge distribution in molecular recognition. The poor guest insertion of hexafluorobenzene (C_6_F_6_) into the perfluorinated complex highlighted the impact of electrostatic repulsion between similarly positive quadrupole moments. The gas adsorption studies further demonstrated differences in the kinetics and adsorption behavior of CO_2_, C_2_H_*n*_ (*n* = 2, 4, 6), and aromatic vapors, underscoring the importance of quadrupole design. These findings provide a rational framework for the development of advanced host–guest materials tailored for selective adsorption and separation applications.

## Abstract 2

A fully fluorinated β-diketonate Cu(ii) complex enhances CO_2_ and benzene adsorption *via* quadrupole interactions. Despite structural similarity, it exceeds the partially fluorinated counterpart, emphasizing charge distribution's role in selectivity.

## Graphical abstract

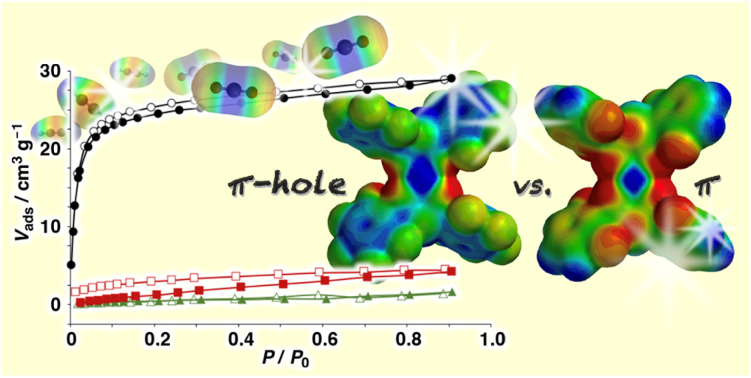

## Introduction

Environmental movements to combat global warming require cost-effective methods for CO_2_ capture. These efforts have become increasingly critical as global emissions continue to rise, primarily resulting from industrial processes and energy production.^1,2^ Innovative materials and strategies are being actively explored to address these challenges. Various classes of porous materials have been investigated, including metal–organic frameworks (MOFs) and porous organic frameworks (POFs).^3–8^ These materials offer high surface areas, controllable pore sizes, and numerous molecular engineering pathways to tune and precisely control host–guest interaction strength through chemical modifications to their molecular building blocks. Size-based separation is currently the most effective method; however, it is not efficient for molecules with similar morphologies, such as CO_2_ and C_2_H_2_, which share rod-like shapes and sizes of 3–5 Å.^9,10^ Industrial gas streams often contain mixtures of these molecules, necessitating advanced technologies to achieve high purity.^11,12^ This limitation has driven the development of alternative approaches that go beyond size-based separation methods. In addition to their similar size and shape, CO_2_ and C_2_H_2_ are non-polar molecules with comparable equilibrium sorption parameters and related physicochemical properties.^13,14^ Therefore, a more rational approach to CO_2_/C_2_H_2_ separation is required. One promising avenue involves leveraging subtle differences in electronic properties, such as quadrupole moments, to achieve selective recognition.

Microporous compounds with cross-sectional areas of 4–6 Å, suitable for small molecules, have been reported to exhibit high levels of selective hydrocarbon adsorption.^15^ These findings highlight the importance of precisely tuned pore environments, as even slight variations in pore size and chemistry can significantly affect selectivity. Conventional molecular recognition processes often favor C_2_H_2_ over CO_2_ because aromatic host frameworks^16–20^ typically interact preferentially with the positive quadrupole moment of C_2_H_2_ (+21.1 × 10^−40^ C m^2^)^21^ rather than the negative quadrupole moment of CO_2_ (negative, −14.3 × 10^−40^ C m^2^).^22^ This interaction is likely influenced by the negative quadrupole moment of aromatic centers, such as that observed in the benzene molecule (−29.0 × 10^−40^ C m^2^).^23^ Building on this concept, the design of host frameworks with tailored electronic properties, guided by quadrupole interactions, has become a focal point for achieving challenging separations involving non-polar molecules. Selective recognition of CO_2_ remains essential from both practical and theoretical perspectives. One potential solution is to invert quadrupole moments to create host materials^24,25^ that preferentially interact with CO_2_. This strategy represents a shift in molecular recognition, moving beyond size-based mechanisms to emphasize electrostatic interactions.

**Scheme 1:**
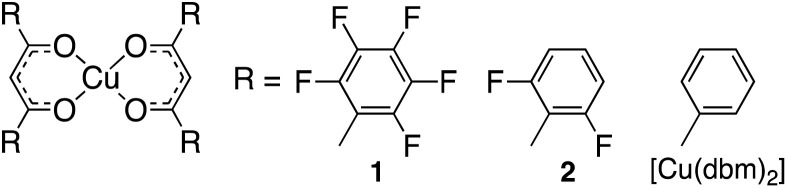


In this study, we investigated the adsorption of CO_2_ and benzene molecules in the non-porous crystals of three β-diketonate Cu(ii) complexes (Scheme 1),^26^ each with varying degrees of fluorination on the phenyl groups. We have previously reported that a fully fluorinated complex 1 acts as a non-porous adaptive crystal (NAC),^27–30^ encapsulating aromatic molecules^31^ and small gases.^32^ Such encapsulation behavior underscores the versatility of NACs, enabling a direct comparison between homogeneous intermolecular interactions among host molecules and heterogeneous interactions between host and guest molecules. This highlights the selective adsorption capability of NACs, particularly for target substances. Additionally, the adsorption adaptability of NACs for non-polar guest molecules with weak intermolecular interactions provides unique insights distinct from conventional porous materials. Steric effects arising from the size of fluorine should be considered, given fluorine's van der Waals radius is approximately 1.2 times larger than hydrogen.^24^ Therefore, this study examines the molecular recognition behavior of structurally similar compounds, such as C_6_F_5_ and *o*-C_6_H_3_F_2_ groups (1 and 2, respectively),^31,33^ which share steric properties but differ in quadrupole moments.^34–36^ Fluorination modifies the electric quadrupole moments of the host framework, introducing electron deficiency and enabling interactions with guest molecules bearing opposite (electron-rich) quadrupole moments. This broadens the potential for capturing CO_2_, with an axially negative quadrupole moment.

## Results and discussion

### Preparation and structural information

**Fig. 1:**
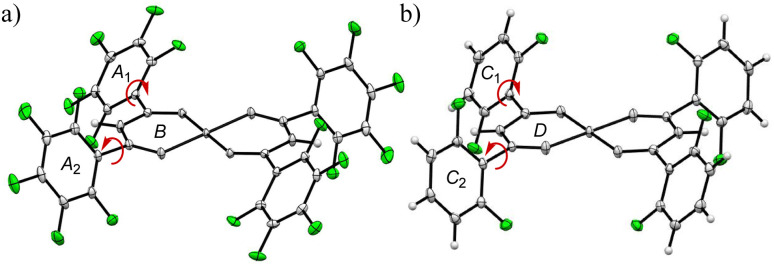
Twisted orientations of the phenyl rings and the coordination of six membered rings of (a) 1 and (b) 2, which were obtained from the single crystallographic data of 1 (CCDC 1850163) and 2 (CCDC 895496): fluorine, green; the other atoms, white.^31,33^

To understand their guest-recognition behaviors, fully fluorinated complex 1 with C_6_F_5_ groups, partially fluorinated complex 2 with *o*-C_6_H_3_F_2_ groups, and the non-fluorinated [Cu(dbm)_2_] (dbm = dibenzoylmethanido^–^) were prepared. The complex motifs were selected as a notable target because dbm is a well-known ligand that forms neutral complexes when coordinated with divalent metal ions. Each complex was prepared using Cu(ii) ions and the corresponding β-diketonate ligands, as previously reported protocols.^26,31^ This characteristic minimizes ionic intermolecular interactions within the crystal structure, allowing a clearer focus on other molecular interactions. Crystallographic and molecular structure analyses revealed that complexes 1 and 2 adopt square-planar coordination geometries with some degree of distortion (Fig. 1). The structural overlay of C, O, and Cu atoms (excluding H and F atoms) of the two complexes shows a close resemblance, with an r.m.s. deviation of 0.142 Å, making them suitable targets for discussing the structural properties and the effects of fluorine substitution (Fig. S1 and S2†). The dihedral angles between the two phenyl groups and the six-membered ring of the coordination plane are similar for complexes 1 and 2, due to the steric hindrance at the *ortho* positions. In complex 1, the dihedral angles are 41.79° and 59.98° for rings A_1_–B and A_2_–B, respectively (Fig. 1a),^33^ while in complex 2, they are 64.18° and 68.81° for rings C_1_–D and C_2_–D, respectively (Fig. 1b).^31^ In contrast, the dihedral angles for [Cu(dbm)_2_] are significantly smaller, at 0.57° and 10.50° for the two rings.^37^ Both crystals 1 and 2 have been confirmed to be molecular crystals without voids through Mercury's void space analysis. However, to predict potential molecular recognition pathways, we calculated the regions where voids are most likely to form. The results suggested that in both cases, potential void spaces are located between the coordination planes of the Cu(ii) ions, specifically above the six-membered CuO_2_C_3_ rings and enclosed by fluorophenyl groups (Fig. S3†). Hirshfeld analysis also indicated that in crystal 2, intermolecular F⋯H/H⋯F interactions are present at the ligand sites, while the predicted void space is oriented toward the fluorophenyl planes rather than their edge regions (Fig. S4 and S5†).

### Quadrupole moments

The quadrupole moments for the principal axis (*Q*_*zz*_) and electrostatic potentials (ESPs) provide complementary insights into the electronic environments of the studied complexes. *Q*_*zz*_ values quantify the electron density distribution along the principal axis, while ESPs visualize the electrostatic potential surrounding the molecule, highlighting regions of electron richness or deficiency. These properties are critical for understanding host–guest interactions, as they determine the compatibility of the host's electronic environment with the characteristics of the guest molecules. The experimental quadrupole moments referenced in the literature, along with the ESPs calculated using density functional theory (DFT)^38^ at the ωB97X-D/6-31G* level for related compounds, are summarized in Table 1. The degree of fluorination significantly influences both *Q*_*zz*_ and ESPs, altering the electronic structure of the aromatic rings. Perfluorination enhances the electron-withdrawing effect, shifting the *Q*_*zz*_ values from negative to positive. For example, *Q*_*zz*_ values (×10^−40^ C m^2^) clearly depend on the degree of fluorination, as reported by A. Vela *et al.*:^34^ −28.6 (benzene) < −7.7 (1,4-difluorobenzene) < +1.9 (1,3,5-trifluorobenzene) < +12.3 (1,2,4,5-tetrafluorobenzene) < +31.9 (hexafluorobenzene). The quadrupole values for CO_2_ and C_2_H_*n*_ hydrocarbons {*n* = 2 (acetylene), 4 (ethylene), 6 (ethane)},^21,35,36^ which have a rod-like morphology, must account for the orientation of each axis (*Q*_*xx*_, *Q*_*yy*_ and *Q*_*zz*_).^39^ This shift in electronic properties directly correlates with the increased electrophilicity of the Cu(ii) centers in complex 1, as indicated by the ESP maps. These features collectively suggest that perfluorinated aromatic systems are well-suited for interactions with negative quadrupolar molecules like CO_2_ and benzene.

**Table 1:** Experimental quadrupole moments for three axis (10^−40^ C m^2^), and calculated ESPs at the centroid of molecule along the *Q*_*zz*_ direction (kJ mol^−1^)

	Formula	*Q*_*xx*_	*Q*_*yy*_	*Q*_*zz*_	ESP^b^
Benzene^23,24^	C_6_H_6_	+14.5	+14.5	−29.0	−94
1,3,5-Trifluorobenzene^36^	C_6_H_3_F_3_	−0.87	−1.0	+1.9	+5.0
Hexafluorobenzene^23,24^	C_6_F_6_	−15.8	−15.8	+31.7	+97
Carbon dioxide^22^	CO_2_	+7.1	+7.1	−14.3	−67
Acetylene^21^	C_2_H_2_	−10.6	−10.6	+21.1	+149
Ethylene^36,39^^,^^a^	C_2_H_4_	−10.5	+4.9	+5.6	+18
Ethane^35^	C_2_H_6_	+1.1	+1.1	−2.2	−20

a Ethylene shows three ESP values of −90, +15, and +18 kJ mol^−1^ for each *C*_2_ axes of the corresponding *Q*_*xx*_, *Q*_*yy*_, and *Q*_*zz*_.

b ESP is calculated at *C*_g_ of *Q*_*zz*_.

**Fig. 2:**
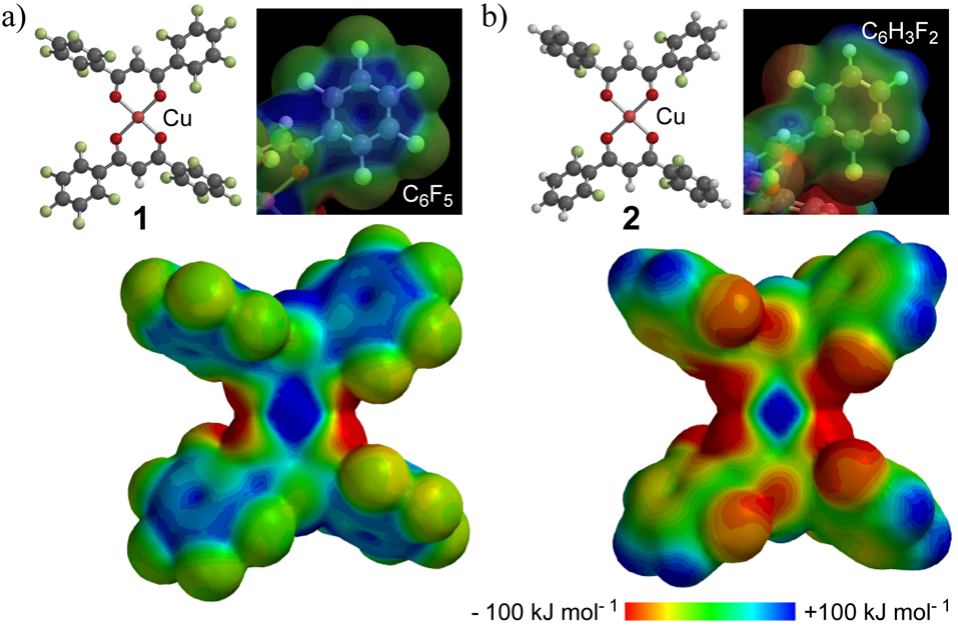
The energy potential maps of (a) 1 and (b) 2: the color of potential is shown between −100 kJ mol^−1^ (red) to +100 kJ mol^−1^ (blue). IsoValue is 0.002.

To understand the guest recognition behavior of fluorinated crystals of 1 and 2, ESP calculations were performed. The ESP maps were generated using DFT based on the atomic coordinates from experimentally determined crystal structures. The ESPs of each metal complex ranged from −159.01 to +206.41 kJ mol^−1^ for 1, and from −224.69 to +127.76 kJ mol^−1^ for 2 (Fig. 2 and Table 2), indicating a strong positive shift in the overall structure of 1 due to perfluorination. The highest ESP, indicated by the blue region (electron-poor), is located on the Cu atom and at the center of the pentafluorophenyl rings in 1, and on the Cu atom and at the edge of the difluorophenyl groups in 2. The lowest ESP, shown in red (electron-rich), is found in the intermediate region between O1 and O2 in both complexes, as well as at the *ortho*-positions of the fluorine atoms in 2. Interestingly, the ESPs at the centroid of the two asymmetric pentafluorophenyl groups in 1 are +96 kJ mol^−1^ for ring-A_1_ and +88 kJ mol^−1^ for ring-A_2_, indicating that they retain the electrostatic properties of pentafluorobenzene (+64 kJ mol^−1^). This relationship is also observed in 2, where the ESPs of the difluorophenyl groups are −28 kJ mol^−1^ for ring-C_1_ and −38 kJ mol^−1^ for ring-C_2_, values comparable to that of 1,3-difluorobenzene (−26 kJ mol^−1^). This agreement with the ESPs of fluorophenyl groups is likely due to the twisted conformation of the fluorophenyl substituents, which effectively prevents electron redistribution from the π-conjugated system. These results strongly support the notion that the positive quadrupole moment of the pentafluorophenyl rings in 1 facilitates the recognition of CO_2_ and benzene, both of which are negative quadrupolar molecules, within their crystals. This observation contributes to a rational approach in the design of host–guest systems.

**Table 2:** Selected ESP value of 1, 2, and [Cu(dbm)_2_]

	1	2	[Cu(dbm)_2_]
Highest positive ESP (copper)	+206.41	+127.76	+116.71
Lowest negative ESP (oxygen)	−159.01	−224.69	−199.12
*C*_g_ of aromatic rings	+96, +88	−28, −38	−75, −85
*C*g of six-membered ring	−14, −17	−88, −90	−73, −79

### Gas and vapor adsorption studies

**Fig. 3:**
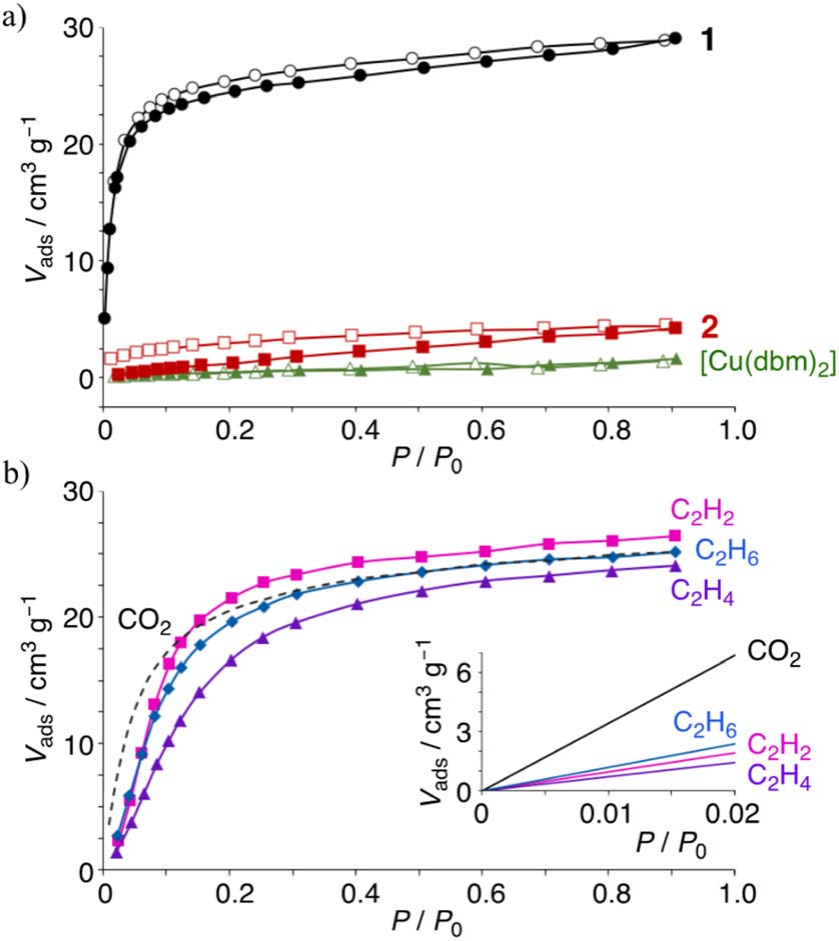
(a) CO_2_ adsorption isotherms at 195 K for: 1, black circles; 2, red rectangles; non-fluorinated [Cu(dbm)_2_], green triangles; filled, adsorption; and open, desorption. (b) C_2_H_*n*_ and CO_2_ adsorption isotherms of 1 at 212 K: C_2_H_2_, pink rectangles; C_2_H_4_, purple triangles; C_2_H_6_, blue lozenges; CO_2_, black line. The internal figure in (b) is approximate curves at extremely low pressure (0–0.02 *P*/*P*_0_).

The gas adsorption/desorption behavior of the complexes was examined using microcrystalline powder samples, activated by heating at 60 °C for 3 h under vacuum. The N_2_ adsorption isotherms for 1 and 2 are type-III, yielding 6.97, 1.02, and 0.54 cm^3^ g^−1^ for 1, 2, and [Cu(dbm)_2_], respectively, at 0.91 *P*/*P*_0_ (Fig. S6†). The CO_2_ adsorption isotherms are shown in Fig. 3a. The fully fluorinated complex 1 adsorbed the highest amount of CO_2_, reaching 29.0 cm^3^ g^−1^ (1.13 mol mol^−1^), compared to 4.24 cm^3^ g^−1^ (0.12 mol mol^−1^) for 2 and 1.58 cm^3^ g^−1^ (0.04 mol mol^−1^) for [Cu(dbm)_2_] at 0.91 *P*/*P*_0_. Complex 1 exhibits a type-I isotherm, with a steep uptake at low *P*/*P*_0_, likely due to enhanced host–guest interactions within the crystal cavities. This corresponds to a 1 : 1 host–guest stoichiometric ratio. Despite the similar steric hindrance from *ortho*-substituted fluorine atoms in 2, which creates comparable axial space around the metal ion (Fig. 1), only minimal CO_2_ uptake was observed. This suggests that the molecular recognition in 1 is primarily driven by its large positive ESP and quadrupole moment. In contrast, the difluorophenyl groups in 2 has a small and negative ESP and quadrupole, which likely prevents CO_2_ uptake. The surface area (*V*_max_/cm^3^) and equilibrium constant (*K*/kPa) obtained by the Langmuir method are 13 and 15 times greater, respectively, for complex 1 than for 2. The temperature dependence of CO_2_ uptake for 1 was also studied (Fig. S7†). The amount of CO_2_ decreased with increasing temperature, as is commonly observed in other porous materials; however, at 298 K, at least 3.9 cm^3^ g^−1^ (0.15 mol mol^−1^ at 0.91 *P*/*P*_0_) of CO_2_ still remained adsorbed. The relatively steep uptake at low *P*/*P*_0_ and low temperatures (<229 K) suggests a strong contribution of quadrupole interactions to adsorption in these non-porous molecular crystals.

Given these results, the uptake of C_2_H_*n*_ hydrocarbons was studied, as hydrocarbon-rich feedstock contains some CO_2_, making its separation, especially from similarly sized components like C_2_H_2_, challenging. The physisorption isotherms for C_2_H_*n*_ hydrocarbons follow a type-I profile (Fig. 3b), although they do not align with expected values at low pressures. The volumes of physisorbed hydrocarbons are 26.4, 24.0, and 25.1 cm^3^ g^−1^ for C_2_H_2_, C_2_H_4_, and C_2_H_6_, respectively, at 0.91 *P*/*P*_0_ and 212 K. These maximum adsorbed amounts are close to the CO_2_ adsorption of 25.2 cm^3^ g^−1^, and the number of C_2_H_*n*_ molecules adsorbed by 1 corresponds to a 1 : 1 stoichiometric ratio. However, the amount of adsorbed CO_2_ is significantly higher at *P*/*P*_0_ values below 0.1, indicating a potential pathway for separating CO_2_ from C_2_H_2_. The adsorption of C_2_H_*n*_ hydrocarbons in 1 may be influenced by the negative quadrupole moment along the *Q*_*xx*_ axis on each π-plane or by adsorption driven by the negative ESP on the oxygens of the complex.

**Fig. 4:**
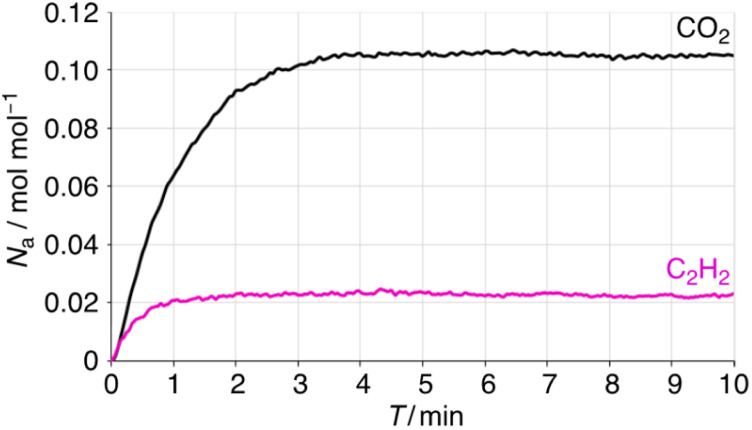
TG studies of weight change ratio of 1 after CO_2_ and C_2_H_2_ gas flow at r.t.

To understand the higher kinetic stability of CO_2_ in 1 at low relative pressures, thermogravimetric (TG) studies were performed under CO_2_ and C_2_H_2_ gas flow at r.t. (Fig. 4). The results show a different rate of weight change over time for CO_2_ and C_2_H_2_, with the weight% and inserted amounts as follows: CO_2_ (0.53%; 0.11 mol mol^−1^) > C_2_H_2_ (6.7 × 10^−2^%; 2.2 × 10^−2^ mol mol^−1^), C_2_H_4_ (5.4 × 10^−2^%; 1.7 × 10^−2^ mol mol^−1^) ≫ C_2_H_6_ (6.4 × 10^−4^%; trace), as shown in Fig. 3 and S8.† Saturation for C_2_H_2_ in 1 was reached within 2 min, while CO_2_ took 4 min under the corresponding gas flow, indicating that CO_2_ has a higher gas intake per minute. This difference explains the higher CO_2_ uptake observed at low *P*/*P*_0_ and demonstrates that fluorination is an effective strategy for CO_2_ capture in non-porous crystals. The very poor insertion of C_2_H_6_ at r.t. suggests that the driving force and host–guest interactions are primarily governed by π-interactions between flat molecules, with minimal effective overlap between the quadrupole moment directions.

**Fig. 5:**
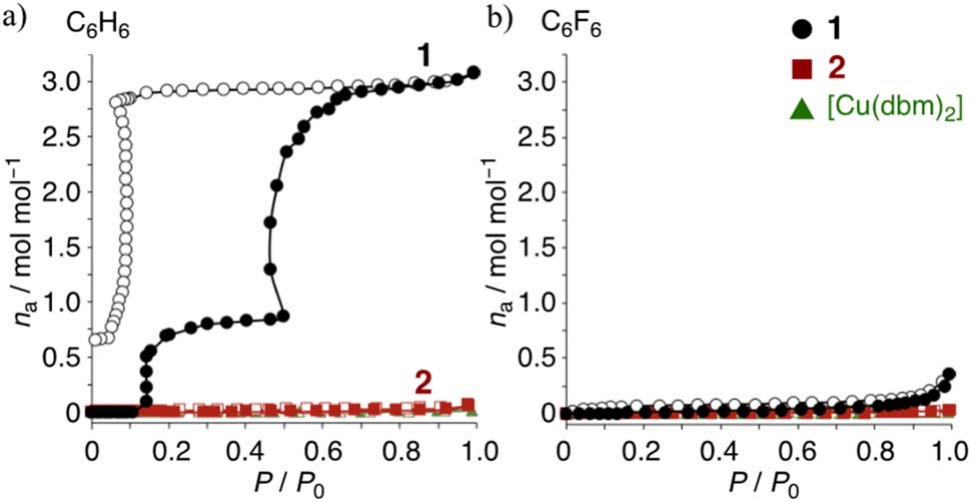
(a) Benzene and (b) hexafluorobenzene adsorption isotherms at r.t. for: 1, black circles; 2, red rectangles; non-fluorinated [Cu(dbm)_2_], green triangles; filled, adsorption; open, desorption.

Similar phenomena were clearly observed for benzene (C_6_H_6_) and hexafluorobenzene (C_6_F_6_) in the adsorption studies. As reported in previous work, three C_6_H_6_ molecules are inserted into the crystal of 1*via* M⋯π and π-hole⋯π interactions,^26^ with one and two equivalents, respectively. The M⋯π interaction refers to the interaction between the Cu center and the π-electron system of benzene, while the π-hole⋯π interaction involves the electrostatic attraction between the electron-deficient region (positive ESP) on a fluorinated aromatic ring and the π-electron system (negative ESP) of benzene. These interactions play a crucial role in stabilizing guest molecules within the host crystal and contribute to the selective adsorption properties observed in this study. The vapor adsorption isotherms of the complexes were examined using activated samples. The C_6_H_6_ isotherms for 1 show an irreversible insertion, unlike 2 and [Cu(dbm)_2_]. In Fig. 5a, one molecule of C_6_H_6_ is inserted at approximately 0.15 *P*/*P*_0_, followed by the insertion of two additional molecules around 0.5 *P*/*P*_0_, resulting in 1·3C_6_H_6_. The desorption isotherm exhibits unique hysteresis and a stepwise release at low pressure. The most stable crystalline state is estimated to be 1·3C_6_H_6_, which aligns with the previously observed crystal structure, suggesting that the remaining benzene molecule around 0.05 *P*/*P*_0_ is likely stabilized by enhanced M⋯π interactions. The lack of guest insertion in 2 clearly indicates that the three-dimensional structure of the molecule does not dominate guest recognition. Instead, the charge distribution on the complex surface plays a crucial role. The C_6_F_6_ isotherms in Fig. 5b show poor guest insertion for all complexes, providing the following insights: (1) electrostatic repulsion, due to the same positive quadrupole moments (*Q*_*zz*_) between the ring-As of 1 and C_6_F_6_, hinders guest insertion; (2) the weak quadrupole moments of the ring-Cs in 2 do not facilitate insertion; (3) while [Cu(dbm)_2_] might have potential for guest recognition, its densely packed planar molecular structure prevents guest insertion. In other word, due to the weak interactions between the pentafluorinated ligands in the crystal, 1 can expand its structure more easily to accommodate guest molecules. In contrast, [Cu(dbm)_2_] is less likely to expand upon crystallization due to M⋯π interactions between the Cu center in the complex and the phenyl ring of the adjusted complexes. Consequently, [Cu(dbm)_2_] cannot adsorb guests even when complementary guests are presented; the inference is supported by the melting points of the complexes: 216 °C for 1 < 321 °C for [Cu(dbm)_2_].^40^

## Experimental

Cu(ii) complexes were obtained as crystalline solids *via* the reaction of the corresponding ligand and CuCl_2_·2H_2_O with NaOCH_3_ in MeOH, with quantitative yields.^26,31^ The solids were activated by heating at 60 °C for 3 hours under vacuum prior to adsorption/desorption measurements. Gas adsorption isotherms were obtained using MicrotracBEL BELSORP Mini/MAX/MAXII, and TG analysis was performed using a TA instruments TGA Q500.

## Conclusions

The fully fluorinated β-diketonate Cu(ii) complex 1 exhibits distinctive gas and vapor adsorption properties in its crystalline state, demonstrating a 1 : 1 CO_2_ and C_2_H_*n*_ (*n* = 2, 4, 6) gas adsorption at 212 K, and a 1 : 3 C_6_H_6_ vapor adsorption at r.t. Notably, the amount of CO_2_ adsorbed by 1 is significantly higher at low pressure (<0.1 *P*/*P*_0_), which is attributed to quadrupole interactions facilitated by the extended positive ESPs on the Cu(ii) center and the pentafluorophenyl rings. This behavior highlights the importance of tailored electronic environments in achieving selective host–guest interactions. The stepwise insertion and release process observed for C_6_H_6_ vapor, characterized by hysteresis, reflects the contributions of M⋯π and π-hole⋯π interactions^41–43^ in stabilizing benzene molecules within the crystal structure. In contrast, the poor guest insertion of C_6_F_6_ into complex 1, caused by electrostatic repulsion between similarly positive quadrupole moments, highlights the importance of charge distribution in molecular recognition. Gas adsorption studies demonstrate the superiority of complex 1 over partially fluorinated complex 2 and non-fluorinated [Cu(dbm)_2_]. The type-I isotherm observed for CO_2_ and the steep uptake at low pressures reflect enhanced host–guest interactions driven by the large positive quadrupole moments in complex 1. This trend, which is absent in 2 and [Cu(dbm)_2_], highlights the critical role of fluorination in enabling selective adsorption. The enhanced affinity for CO_2_ and C_6_H_6_ in fluorinated compounds provides a novel framework for designing materials with complementary adsorption characteristics. These findings open avenues for further exploration of quadrupole modulation in gas separation technologies.

## Data availability

The data supporting the findings of this study, including preparation methods, structural information, and physical properties, are available in the main article and its ESI.† Further inquiries regarding specific experimental details can be directed to the corresponding author. Additional information is available at the following sources: crystallographic data: data for compounds 1 and 2 was obtained from the Cambridge Crystallographic Data Centre under deposition numbers 1850163 and 895496, respectively. These can be obtained from the CCDC website (https://www.ccdc.cam.ac.uk/structures/). Preparation of 1: DOI: https://doi.org/10.1039/B617808A. Preparation of 2: DOI: https://doi.org/10.1039/C4CE01243G. Quadrupole moments: refer to ref. 16 and 26–28, and the Computational Chemistry Comparison and Benchmark DataBase (CCCBDB) (https://cccbdb.nist.gov/introx.asp). Adsorption data: included as part of the ESI.†

## Author contributions

Y. I. prepared the samples and conducted the gas studies. T. K. performed theoretical studies based on the crystal data and vapor adsorption. Y. I. (2) and H. U. assisted gas and vapor adsorption measurements, respectively, and ensured the data reproducibility. S. U. performed the TG analysis and contributed the absorption project. K. K. and M. N. provided support for the adsorption project. A. H. supervised the project and wrote the manuscript. All authors contributed to discussions and to finalizing the manuscript.

## Conflicts of interest

There are no conflicts to declare.

## Supplementary Material

RA-015-D4RA08463B-s001
