# Supplementary material for: Boosting CO2 and benzene adsorption through π-hole substitution in β-diketonate Cu(ii) complex within non-porous adaptive crystals
Source: RSC Adv. 2025 Feb 25;15(8):6184–90. doi: 10.1039/d4ra08463b (PMC11851097; doi:10.1039/d4ra08463b)
Supplement: RA-015-D4RA08463B-s001 [file RA-015-D4RA08463B-s001.pdf]

Supplementary Information (SI) for RSC Advances.
This journal is © The Royal Society of Chemistry 2025

Electronic Supplementary Information

Boosting CO_2 and Benzene Adsorption through π-Hole Substitution in β-Diketonate Cu(II) Complex within Non-Porous Adaptive Crystals

Y. Ikumura, T. Kawasaki, Y. Ishida, H. Usui, S. Uchida, K. Kamata, M. Nomura, A. Hori*
*Correspondence author (e-mail: ahori@shibaura-it.ac.jp)

Table of Contents

S1. Crystallographic information of **1** and **2** ····· p. 1
S2. Hirshfeld surface analysis of **1** and **2** ····· p. 3
S3. Adsorption studies of **1** and **2** ····· p. 6

S1. Crystallographic information of 1 and 2

The crystallographic data of **1** and **2** were retrieved from CCDC1850163 (J. M. Crowder, et al., *Polyhedron*, 2019, **157**, 33) and CCDC895496 (A. Hori, et al., *CrystEngComm*, 2014, **16**, 8805), respectively, for comparison of their structures and supramolecular associations.

1) Packing structures of **1** and **2**

a) **1**

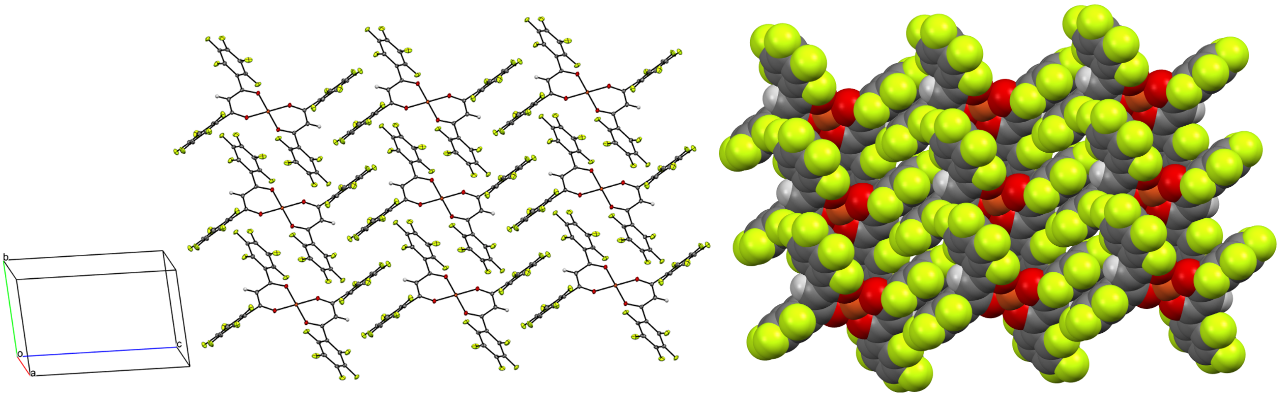

b) **2**

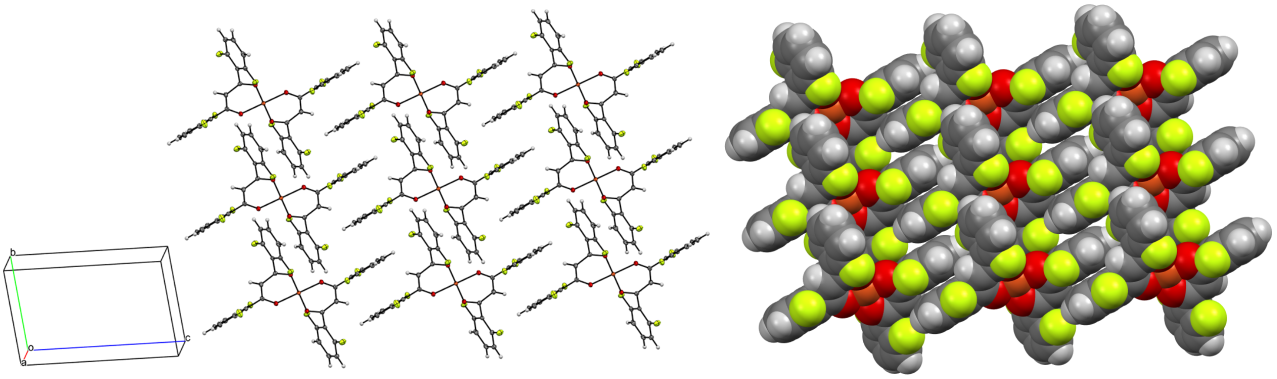

Figure S1. Crystal packing structures of a) **1** and b) **2**.

2) Structural overlay of **1** and **2** using Mercury

The structural overlay of **1** and **2**, based on a) CuO_4 (metal center), b) CuO_4C_6 (six-membered ring coordination), and c) CuO_4C_{30} (excluding H and F atoms), along with the corresponding r.m.s. deviations from single-crystal data, clearly demonstrates their structural similarity.

a) b) c)

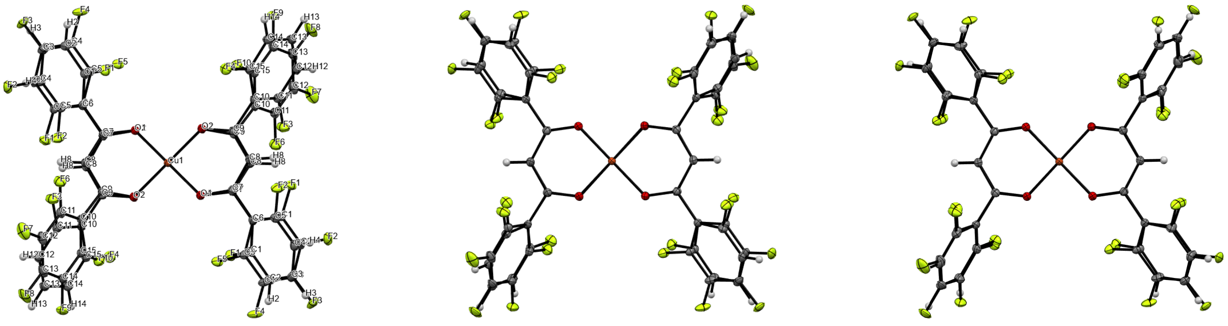

CuO_4 (metal center) — r.m.s 0.0041

CuO_4C_6 (coordination 6-membered rings) — r.m.s 0.102

CuO_4C_{30} (without H and F atoms) — r.m.s 0.142

Figure S2. Structural overlays and r.m.s deviations of **1** and **2**.

3) Estimated void space and corresponding volumes of **1** and **2** using Mercury

The unoccupied spaces (voids) within the crystal unit cells were determined by evaluating whether a spherical probe of a specified radius could fit into them. A probe radius is 1.2 Å for the van der Waals radius of hydrogen. The potential guest incorporation site is presumably located between the two coordination planes, attributed to the steric influence of bulky fluorophenyl groups.

a) **1** b) **2**

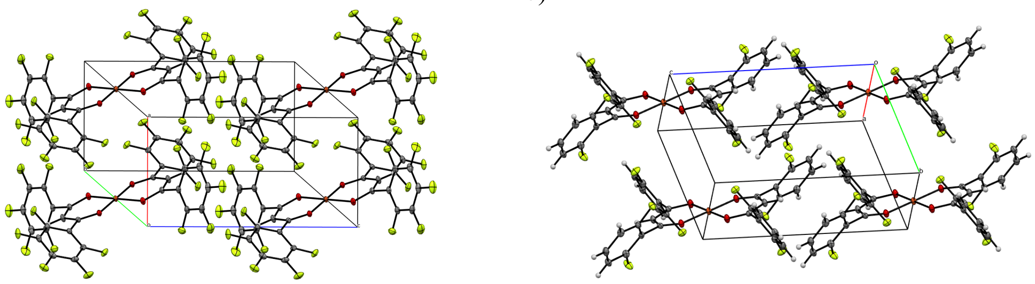

Probe 1.0 Å 0% 0%

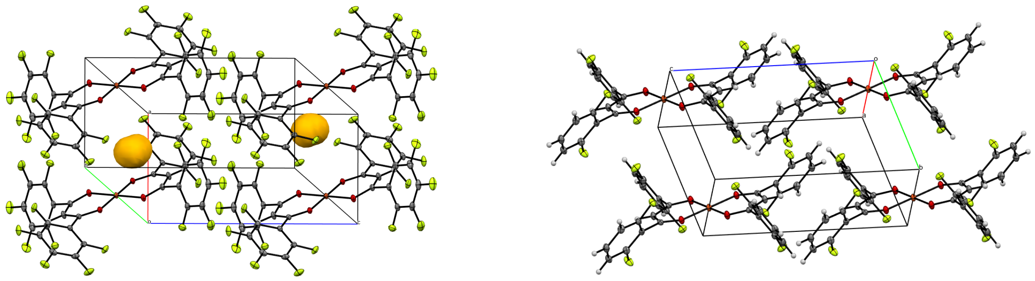

Probe 0.9 Å 1.3% (8.92 Å^3) 0%

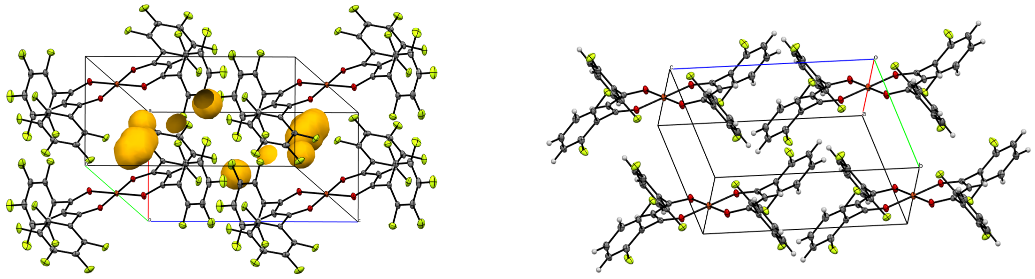

Probe 0.8 Å 3.5% (24.21 Å^3) 0%

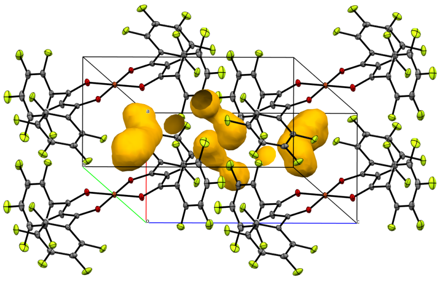 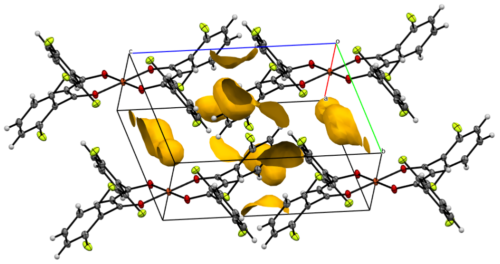

Probe 0.7 Å 5.9% (41.23 Å^3) 5.3% (32.61 Å^3)

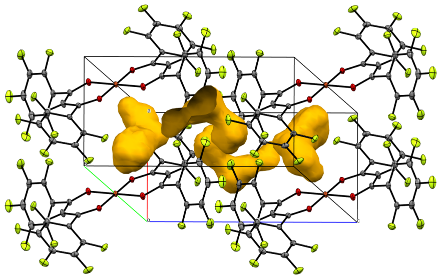 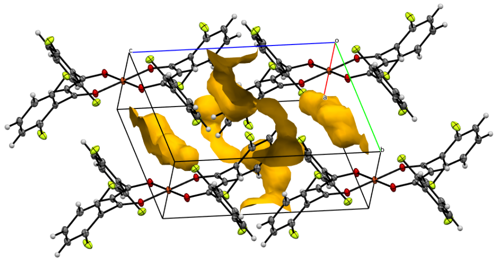

Probe 0.6 Å 8.0% (55.95 Å^3) 7.7% (47.30 Å^3)

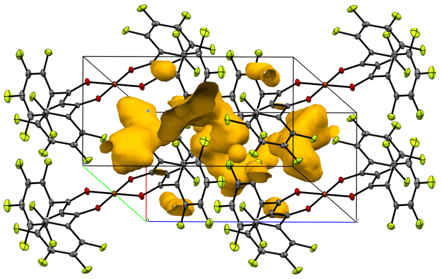 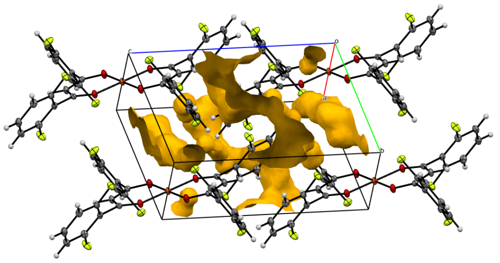

Probe 0.5 Å 10.9% (76.54 Å^3) Void volume 11.1% (68.17 Å^3)

Figure S3. Spherical probe analysis and corresponding void volume of **1** and **2**.

S2. Hirshfeld surface analysis of 1 and 2.

Hirshfeld Surface (HS) analysis was performed to evaluate the contribution of intermolecular interactions. Fingerprint plots were generated to visualize intermolecular interactions in the HS: the horizontal axis (d_i) represents the distance from an atom inside the surface to the nearest external atom, while the vertical axis (d_e) indicates the distance from an external atom to the closest internal atom.

1) Fingerprint plots of **1**

Cu (in)···All (out) 1.4% Cu (in)···F (out) 1.4% Cu (in)···C, Cu, H, O (out) 0.0%

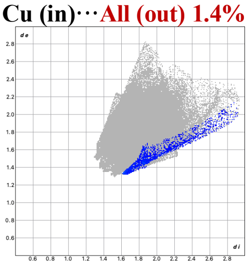 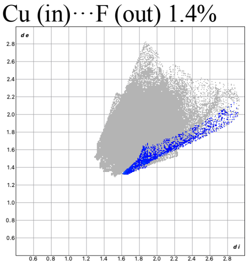

C (in)···All (out) 20.7% C (in)···C (out) 6.0% C (in)···F (out) 13.9% C (in)···O (out) 0.8%

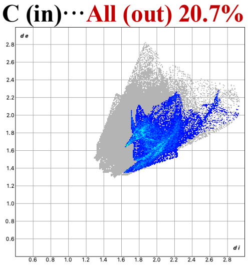 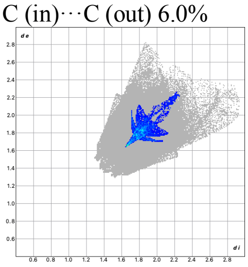 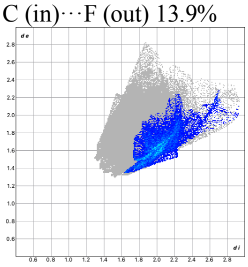 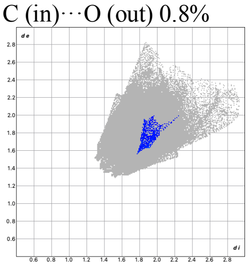

C (in)···Cu, H (out) 0.0%

F (in)···All (out) 66.6% F (in)···C (out) 11.5% F (in)···Cu (out) 1.3% F (in)···F (out) 43.9%

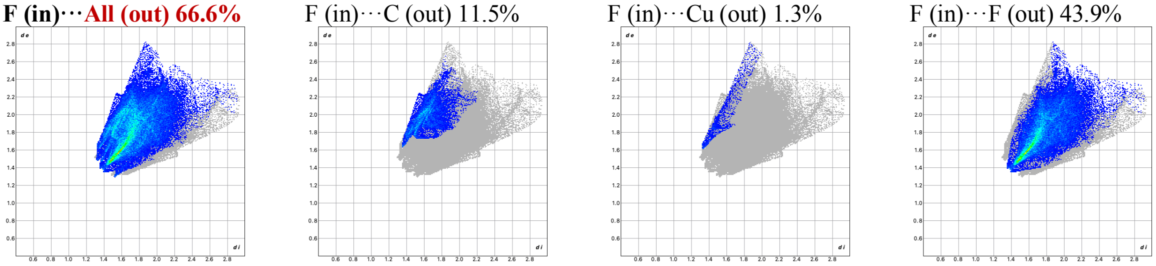

F (in)···H (out) 4.3% F (in)···O (out) 5.6%

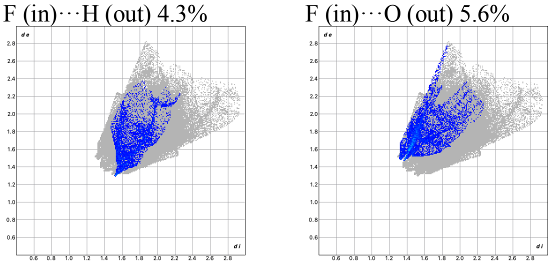

H (in)···All (out) 4.2% H (in)···F (out) 4.2% H (in)···C, Cu, H, O (out) 0.0%

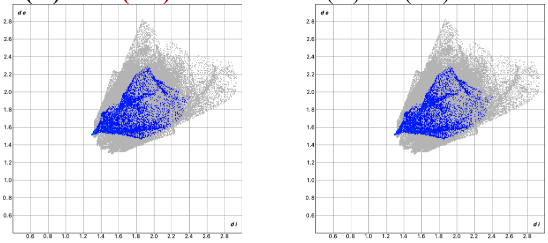

O (in)···All (out) 7.1% O (in)···C (out) 0.8% O (in)···F (out) 6.3% O (in)···Cu, F, O (out) 0.0%

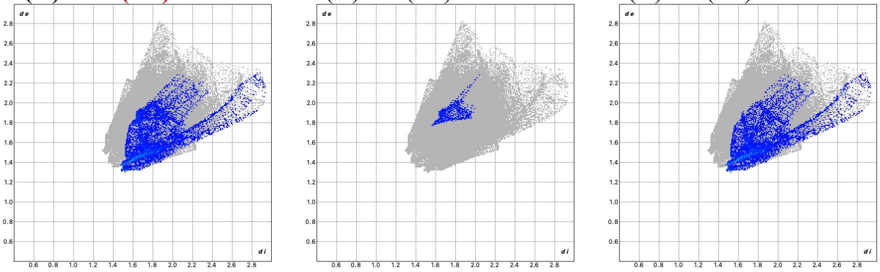

Figure S4. Fingerprint plots for **1** showing d_e and d_i ranging from 0.6 to 3.0 Å for all atoms.

2) Fingerprint plots of **2**

Cu (in)···All (out) 1.5% Cu (in)···F (out) 0.9% Cu (in)···H (out) 0.6% Cu (in)···C, Cu, O (out) 0.0%

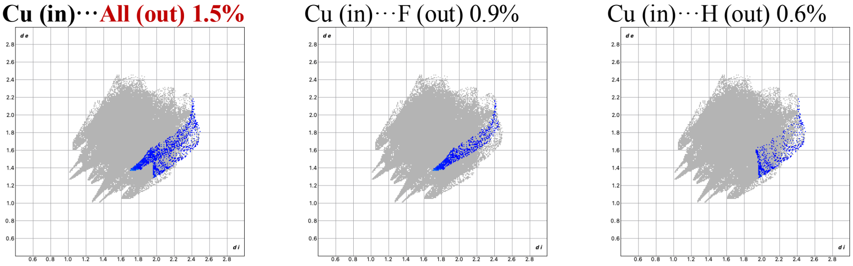

C (in)···All (out) 21.7% C (in)···C (out) 7.5% C (in)···F (out) 3.6% C (in)···H (out) 10.6%

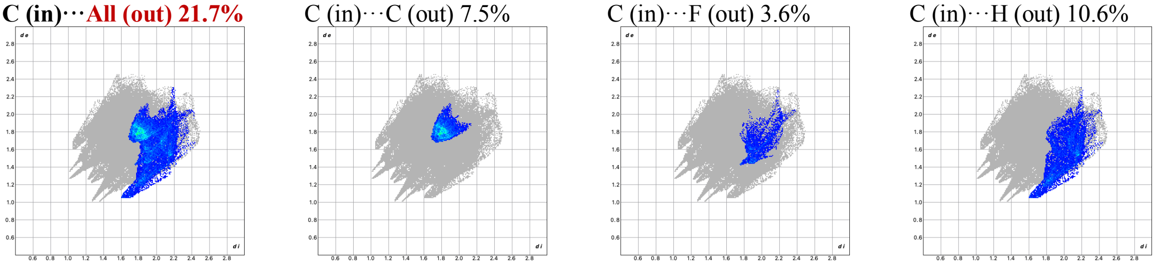

C (in)···O (out) 0.1%; C (in)···Cu (out) 0.0%

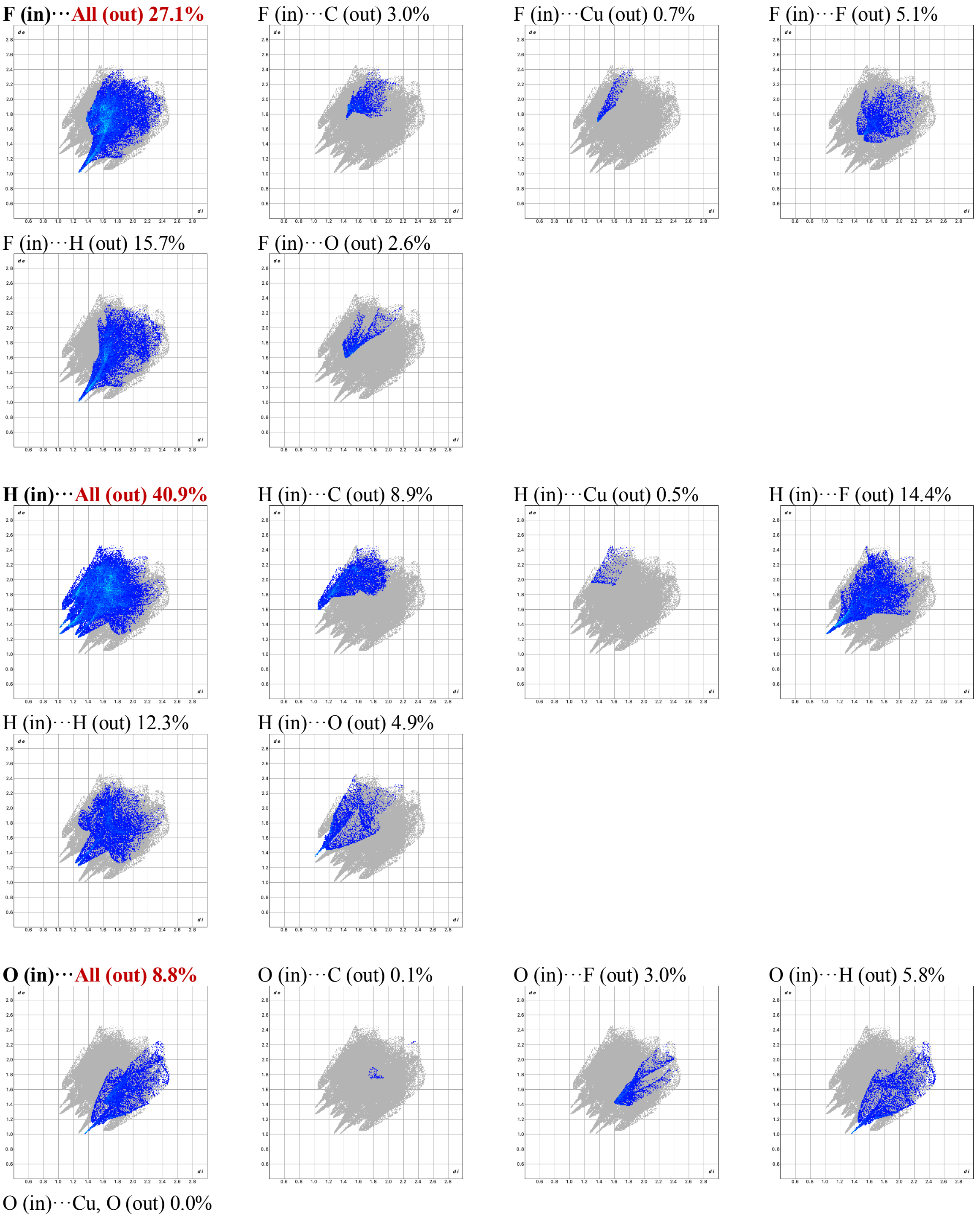

Figure S5. Fingerprint plots for **2** showing d_e and d_i ranging from 0.6 to 3.0 Å for all atoms.

S3. Adsorption studies of 1 and 2

Adsorption behavior of N_2, CO_2, C_2H_2, C_2H_4, and C_2H_6 was examined using BelsorpMax and TGA.

1) Adsorption data for **1**, **2**, and [Cu(dbm)$_2$]

The N_2 adsorption isotherms for **1** and **2** follow type-III behavior, with adsorption capacities of 6.97, 1.02, and 0.54 cm^3 g^{-1} for **1**, **2**, and [Cu(dbm)$_2$], respectively, at 0.91 P/P_0. These results characterize them as non-porous adaptive crystals (NACs). The CO_2 adsorption isotherms for **1** and **2** follow type-I and III behavior, respectively, with 29.0 cm^3 g^{-1} for **1** and 4.24 cm^3 g^{-1} for **2**, at 0.91 P/P_0. These results suggest a strong insertion pathway for CO_2 in the fully fluorinated complex **1**.

a) N_2 b) CO_2

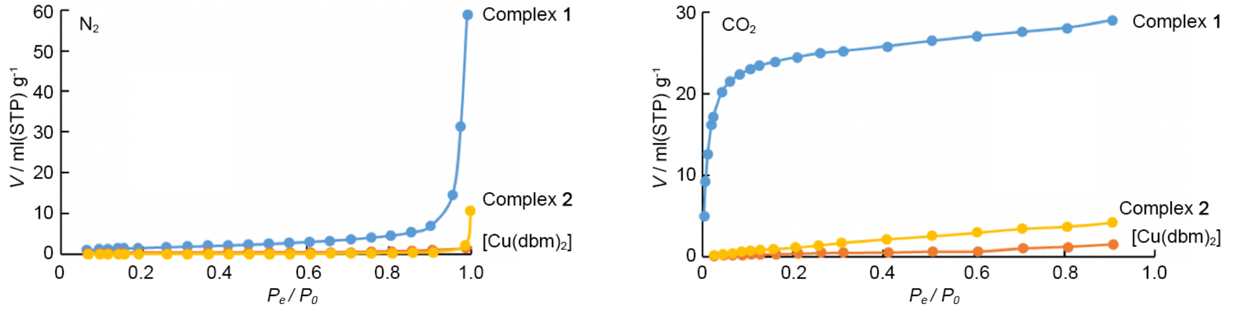

Figure S6. Adsorption isotherms of a) N_2 at 77 K and b) CO_2 at 195 K for **1** (blue), **2** (yellow), and non-fluorinated [Cu(dbm)$_2$] (orange).

2) Adsorption data for **1** and [Cu(dbm)$_2$]

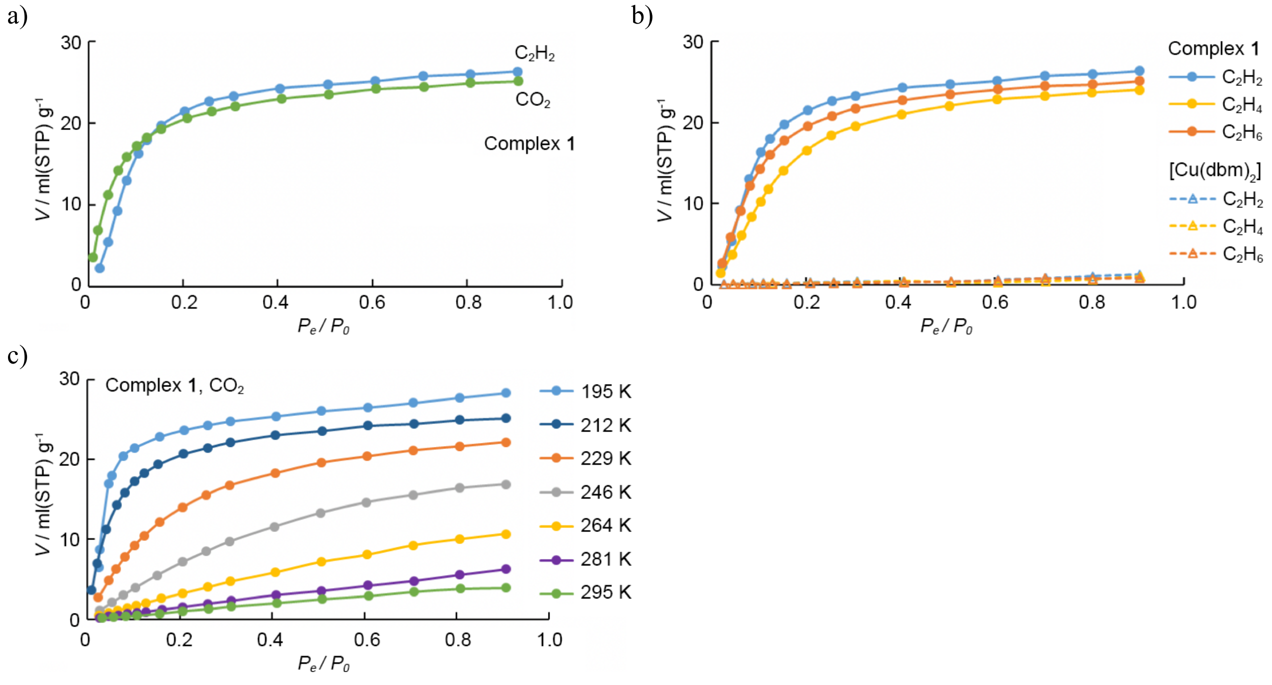

Figure S7. Adsorption isotherms of a) CO_2 and C_2H_2 for **1** at 212 K, b) C_2H_2, C_2H_4, and C_2H_6 for **1** and [Cu(dbm)$_2$] at 212 K, and c) temperature dependent CO_2 adsorption for **1**.

3) Thermogravimetric (TG) analysis of **1** at room temperature

a)

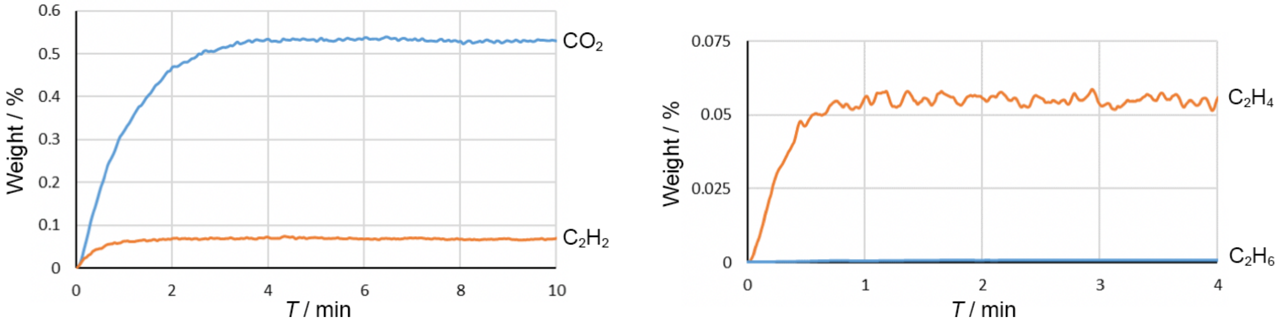

b)

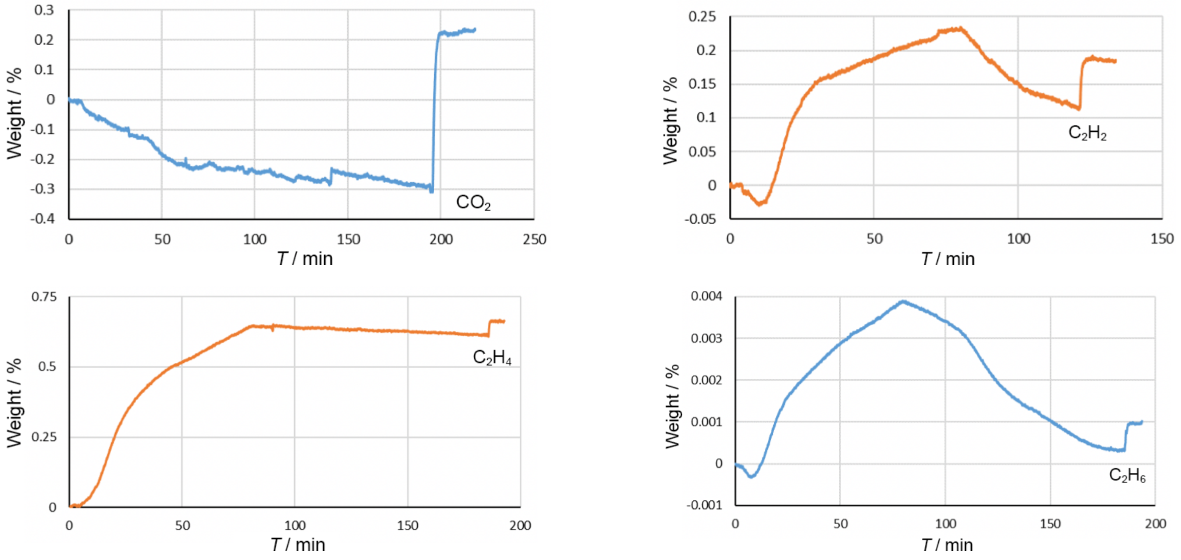

Figure S8. a) Weight comparisons and b) the corresponding experimental results for **1** after gas flow.
